# Minimally Cooked Potato Improved Glycemic Response Across Two Meals and Insulin Sensitivity of Rice–Potato Mixed Meals: A Randomized Controlled Acute Trial

**DOI:** 10.3390/nu18060973

**Published:** 2026-03-19

**Authors:** Jinjie Wei, Zhihong Fan, Yixiao Deng, Kainan Pan, Ruizhe Shi, Jiahui Hu, Baoyue Liu

**Affiliations:** 1 College of Food Science and Nutritional Engineering, China Agricultural University, 17 Qinghua East Road, Beijing 100083, China; s20233061238@cau.edu.cn (J.W.); 18080298831@163.com (Y.D.); 15268486889@163.com (K.P.); zplasz0813@163.com (R.S.); hujiahui1023@126.com (J.H.); lbyuejune@163.com (B.L.); 2 Key Laboratory of Precision Nutrition and Food Quality, Department of Nutrition and Health, China Agricultural University, Beijing 100193, China

**Keywords:** potato, glycemic response, insulin response, resistant starch, cooking, oral processing, texture, minimal processing

## Abstract

**Objectives:** This study aimed to investigate the possible association among texture, oral processing and starch digestive characteristics of hard-cooked (HP) and soft-cooked (SP) potato samples, as well as their acute postprandial glycemic and insulinemic response, when co-ingested with rice in a meal. **Methods:** HP and SP replaced one-third of rice carbohydrates. Postprandial glycemic and insulinemic responses were measured after test meal ingestion. In vitro experiments evaluated sample physicochemical properties. **Results:** HP retained more resistant starch (RS) and total phenolics than SP. When co-ingested with rice (HP + R), HP elicited more total chews, higher oral sensory exposure time, slower chewing frequency and longer eating duration. HP + R significantly reduced postprandial glucose iAUC, peak glucose and glycemic excursion. SP + R increased glycemic variability despite reduced iAUCglucose. HP + R also lowered iAUCinsulin, peak insulin and insulin resistance index. The hypoglycemic effect did not extend to the second meal, though composite iAUCglucose over 540 min was reduced. **Conclusions:** Partially substituting rice with hard-cooked potatoes may help stabilize postprandial glycemic and insulinemic responses, an effect largely attributable to RS retention.

## 1. Introduction

The global prevalence of diabetes is estimated to affect 783 million people by 2045 and pose a threat to even more individuals with prediabetes, posing a serious health challenge [1]. People with impaired glucose tolerance often experience increased postprandial blood glucose excursions, which can impair the function of pancreatic β-cells [2] and is associated with conditions including polycystic ovary syndrome, obesity, and cardiovascular disease [3,4,5]. Therefore, stabilizing glucose levels through the wise selection of starchy foods is a critical issue in regions with high carbohydrate consumption.

As one of the world’s most important starchy crops [6], potatoes can serve either as a staple food, when baked or steamed, or as an ingredient in many dishes. Regardless of the diverse ways they are consumed, potatoes provide a substantial amount of carbohydrates in diets across most global regions.

Unlike cereals, potatoes are widely recognized as one of the most cost-effective dietary sources of potassium, fiber, vitamin C, and antioxidants. It is reported that the potassium and phenolic contents in potatoes can reach up to 346 mg and 73.87 mg per 100 g, respectively [7,8].

However, existing observational studies have reached inconsistent conclusions regarding the association between potato consumption and gestational diabetes mellitus (GDM) [9], weight changes [10], and mortality [11]. Some studies suggest that total potato intake during early [12] or mid-pregnancy [13] is associated with an increased risk of GDM. Conversely, other studies have found a negative or insignificant association between the glycemic load from starchy vegetables, including potatoes, and GDM risk [14,15]. Similarly, studies examining the relationship between potato intake and type 2 diabetes (T2D) have produced conflicting results. One study identified a significant association between higher consumption of fried potatoes and an increased T2D risk [16], while another study [17] found that participants with a higher overall potato intake had a significantly lower risk of developing diabetes. This inconsistency in findings may be attributed to varying consumption patterns.

When served as a dish (e.g., cooked with meat) or as a snack (e.g., crisps and chips or fried potatoes) in addition to grain staples, potato-based foods can increase the proportion of energy derived from carbohydrates and raise the diet’s glycemic load. However, when potato foods are used as a staple food in replacement of refined cereals, they can significantly increase potassium, vitamin C, and dietary fiber intake without substantially affecting the dietary glycemic load or adding extra calories. This dietary adjustment may improve the potassium-to-sodium ratio and boost antioxidant intake, which helps prevent hypertension and hyperuricemia [18,19].

Furthermore, when examining the association between potato consumption and diabetes risk, variations in cooking methods must be taken into account. A previous study reported that individuals who consume more boiled potatoes have a significantly lower risk of T2D [17]. Another study found that it was the intake of fried potatoes, rather than baked, boiled, or mashed potatoes, that was associated with a higher risk of T2D [16]. Notably, replacing white rice with potato-based foods is generally associated with a lower risk of T2D [16].

The texture of potatoes can vary considerably depending on the cooking method, ranging from firm and crispy to soft and mashed. In Chinese cuisine, potatoes are usually prepared in two ways: stir-frying, which involves brief heating to retain a hard and crispy texture; and stewing, baking, or steaming, which results in a soft texture. These two distinctive textures may induce different glycemic responses. Previous research reported that the glycemic index (GI) of a fully cooked soft textured potato sample was 82.9, while a minimally cooked hard textured potato sample was 56.2, respectively [20].

It has also been confirmed that hard-cooked potatoes can serve as an excellent dietary source of resistant starch (RS). Given the significant role of RS in regulating chronic disease risk and promoting gut health [21,22], it is plausible that partially replacing refined grain staples with lightly processed potatoes may improve postprandial glycemic responses by increasing dietary RS intake.

Furthermore, the oral processing characteristics of food, including eating rate and chewing effort, can be modified by manipulating the texture of food. Enhanced chewing and a slowed eating rate have been reported to be associated with increased secretion of glucagon-like peptide-1 (GLP-1) and peptide YY, as well as improved insulin sensitivity [23].

Although long-term evidence on the glycemic outcomes of differently cooked potato products is lacking, a few acute trials have suggested that improved glycemic responses can be achieved by partially replacing white rice with potato-based foods. A previous study reported that, on an isocaloric basis, consuming a potato preload containing 15 g of available carbohydrates significantly reduced postprandial glycemic response compared with consuming rice alone [20]. However, several scientific questions warrant further investigation. Firstly, can potato substitution mitigate postprandial blood glucose excursions when co-consumed with rice rather than as a preload? Secondly, compared with its low-RS soft-cooked counterpart, will high-RS hard-cooked potato food produce a greater reduction in postprandial glycemic response when consumed as a partial replacement for rice? Previous research suggests that RS in low-glycemic index foods may induce a “second-meal effect” through colonic fermentation and short-chain fatty acid production [24,25]. Therefore, the third question is: if so, will the expected hypoglycemic effect of high-RS hard-cooked potato food extend to subsequent meals? The answers to these questions may provide foundational information for future intervention studies aimed at optimizing potato consumption in dietary glycemic management.

To address these questions, the present study designed an acute, randomized, self-controlled trial to investigate the potential glycemic effects of potatoes cooked to varying degrees, based on isocaloric carbohydrate intake. In the trial, hard-cooked potato (HP) and soft-cooked potato (SP) samples were used to replace one-third of the carbohydrates typically provided by white rice. In vitro experiments were conducted to evaluate the digestive characteristics and textural properties of the potato samples. In addition, to explore the potential association between the food matrix and postprandial glycemic response, oral processing behaviors were also assessed.

In this study, the following hypotheses are proposed: (1) partially substituting white rice with potatoes in a high-glycemic meal would help stabilize postprandial glucose and insulin responses; (2) HP is more effective than SP in improving postprandial glycemic responses; (3) the differences in glycemic and insulin effects between HP and SP may be attributable to their distinct digestible starch fractions and textural properties; and (4) the impact of HP and SP on glycemic responses and satiety may extend to the second meal.

## 2. Materials and Methods

### 2.1. Materials and Instruments

Fresh potatoes (*Solanum tuberosum* L.) were sourced from Zaozhuang, Shandong Province. Romaine lettuce (*Lactuca sativa* var. *longifolia*) was obtained from Kunming, Yunnan Province. Cherry tomatoes (*Lycopersicon esculentum* var. *cerasiforme*) were sourced from Weifang, Shandong Province. All fresh produce was supplied by Beijing Xiangxian Biotechnology Co., Ltd. (Beijing, China). Japonica rice was provided by Harbin Tuanmao Food Co., Ltd. (Harbin, China). Kewpie roasted sesame salad dressing was obtained from Beijing Kewpie Food Co., Ltd. (Beijing, China). Tyson chicken tenderloin (black pepper flavor) was supplied by Tyson East China Food Development Co., Ltd. (Nantong, China). Wangjiadu luncheon meat sausage was produced by Sichuan Wangjiadu Food Co., Ltd. (Meishan, China). Toast bread was provided by Taoli Bread Co., Ltd. (Shenyang, China). Pure milk was sourced from Inner Mongolia Yili Industrial Group Co., Ltd. (Hohhot, China).

The following materials were used for blood sampling and biochemical analyses: disposable blood lancets (Suzhou Scharry Medical Devices Co., Ltd., Suzhou, China); disposable anticoagulant centrifuge tubes (0.5 mL capped conical-bottom tubes, Junlibo Biotechnology Co., Ltd., Beijing, China); human insulin (INS) ELISA kit (Jiangsu Meimian Industrial Co., Ltd., Yancheng, China); amyloglucosidase (Sigma-Aldrich, Shanghai, China); pancreatin (P1625, Sigma-Aldrich, Shanghai, China); sodium acetate buffer (Beijing Kubolai Technology Co., Ltd., Beijing, China); anhydrous ethanol, standard glucose sample, and 3,5-dinitrosalicylic acid (Bellingwei Chemical Reagent Co., Ltd., Beijing, China).

The main instruments employed were: Synergy HT microplate reader (BioTek Instruments, Inc., Winooski, VT, USA); LQ-C20002 balance (Shanghai Yaoxin Electronic Technology Co., Ltd., Shanghai, China); electric rice cooker (MB-FZ4086, Midea, Foshan, China); Supor SY-18YA9061 mini pressure cooker (Supor Living Appliance Manufacturing Co., Ltd., Shaoxing, China); SHA-B constant temperature shaker (Guohua Company, Shenzhen, China); TGL18M benchtop high-speed refrigerated centrifuge (Yancheng Kaite Experimental Instrument Co., Ltd., Yancheng, China); ONE TOUCH Verio test strips and glucometer (Lifescan Medical Devices Co., Ltd., Shanghai, China); Abbott FreeStyle Libre continuous glucose monitor (Abbott Diabetes Care Ltd., Shanghai, China); electronic blood pressure monitor (HEM-7200, OMRON, Dalian, China); SPL6506BM camera (Philips (China) Investment Co., Ltd., Shanghai, China); and TA.XT.plus Texture Analyzer (SMS Company, Godalming, Surrey, UK).

All potatoes used in the experiment were purchased from Xiaoxiang Fresh Supermarket. Before cooking, they were peeled and sliced to a thickness of approximately 0.75 cm. For the hard-cooked potato (HP), the sliced potatoes were added to water at a ratio of 1:0.3 (potato weight:water weight) and cooked in the rice pressure cooker until the rated pressure was reached, followed by a 30 s pressure hold. For the soft-cooked potato (SP), the same pressure cooker was used, but the pressure was maintained for 20 min after reaching the rated pressure. The pressure was released immediately after the cooking time elapsed by pressing the pressure-relief valve. The core temperatures of potato samples immediately after cooked were 57.6 °C (HP) and 77.4 °C (SP). Rice was cooked in the rice cooker using the fast-cooking mode with a water-to-rice ratio of 1.7. Raw potato (RP) was used as a control in physicochemical experiments.

### 2.2. Physical and Chemical Analyses

#### 2.2.1. Basic Nutrient Analysis

Physicochemical composition of starchy vegetables and japonica rice are shown in the Table 1. Moisture content was determined according to Method 1 of GB 5009.3-2016 [26]. Fat content was determined according to Method 2 of GB 5009.6-2016 [27]. Protein content was determined according to Method 1 of GB 5009.5-2016 [28]. Dietary fiber content was determined according to GB 5009.88-2023 [29]. Starch content was determined according to Method 2 of GB 5009.9-2023 [30]. Ash content was determined according to GB 5009.4-2016 [31].

#### 2.2.2. In Vitro Digestibility Assessment

In vitro digestibility was assessed using the method described by Englyst et al., with slight modifications [32]. Raw and cooked samples (1 g each) were mixed separately with 15 mL of sodium acetate buffer solution (pH 5.2, 0.2 M). A composite enzyme solution was prepared using the buffer solution as the solvent, containing 15 U/mL amyloglucosidase and 290 U/mL porcine pancreatic α-amylase. Then, 10 mL of the composite enzyme solution was added to each sample mixture, which was incubated at 37 °C in a constant-temperature water bath shaker at 200 rpm for 2 h. Aliquots (0.5 mL) were collected at 0, 20, and 120 min. Immediately after collection, 4.5 mL of anhydrous ethanol was added to each aliquot to inactivate the enzymes and terminate the digestion reaction. Subsequent measurements were performed using the 3,5-dinitrosalicylic acid (DNS) method. A 0.5 mL portion of the terminated reaction mixture was uniformly mixed with 1 mL of DNS reagent and then boiled in a water bath for 3 min until complete color development was achieved. The mixture was then diluted to 15 mL with deionized water, and the absorbance was measured at 540 nm. Each treatment group was analyzed in quintuplicate.

Glucose standard solutions were prepared at concentrations of 0, 0.2, 0.4, 0.6, and 0.8 mg/mL using glucose reference material. Following the above procedure, 0.5 mL of each standard solution was mixed with 1 mL of DNS reagent, boiled for 3 min in a water bath until fully colored, diluted to 15 mL with deionized water, and the absorbance was measured at 540 nm.

#### 2.2.3. Determination of Total Phenol Contents

A 1.5 g sample of cooked and ground potato was placed into a 50 mL centrifuge tube. Then, 20 mL of 80% methanol solution was added as the extraction solvent. The mixture was homogenized for 3 min, subjected to ultrasonic extraction for 15 min, and then centrifuged at 10,000 rpm at 4 °C for 10 min. This extraction procedure was repeated twice. The supernatants were combined and made up to volume in a 50 mL volumetric flask. Folin–Ciocalteu reagent was diluted tenfold with deionized water. Then, 0.8 mL of the sample solution was mixed with 4 mL of the diluted Folin–Ciocalteu reagent, followed by the addition of 3.6 mL of 7.5% Na_2_CO_3_ solution. The mixture was allowed to react at room temperature, protected from light, for 1 h. The absorbance was measured at 765 nm. Total phenolic content was determined by the Folin–Ciocalteu method and expressed as gallic acid equivalents per 100 g dry weight (mg GAE/100 g DW). Each treatment group was analyzed in quintuplicates.

For the standard curve, 1 mg of gallic acid standard was dissolved in deionized water, shaken thoroughly, and diluted to 100 mL to prepare a standard stock solution. This stock solution was used to prepare gradient standard solutions at concentrations of 0, 20, 40, 60, 80, and 100 μg/mL. Subsequently, 0.8 mL of each standard solution was mixed with 4 mL of diluted Folin–Ciocalteu reagent, followed by the addition of 3.6 mL of 7.5% Na_2_CO_3_ solution. The mixture was allowed to react at room temperature, protected from light, for 1 h. The absorbance of each standard was measured at 765 nm.

#### 2.2.4. Determination of Puncture Characteristics and Shear Characteristics

Puncture characteristic test method: Select slices of cooked vegetables for penetration testing. Take the slices from the middle edible portion and ensure they are approximately 0.5 cm thick. Prior to testing, select vegetable slices that are relatively uniform in size, shape and color, and discard any slices that have been significantly damaged during cooking.

Building upon the Exponent software 6.1.26.0 Sample Project method, modifications were implemented using a P/2 probe and an HDP/90 platform. The test mode was set to compression with the return to start option selected. The pre-test velocity was set to 1.5 mm/s, the test velocity to 1.0 mm/s and the post-test velocity to 15.0 mm/s. Target mode: Distance. Test distance: 5 mm. Trigger type: Force. Trigger force: 5.0 g. Data acquisition rate: 200 pps.

When installing the probe and platform, ensure that the vertical projection of the probe is precisely aligned with the central hole in the platform. Observed parameters: Puncture strength (g).

Puncture location: Place vegetable slices flat on the platform. Find an appropriate testing position 0.5–1 cm from the centre of the slice, avoiding any holes. Select five points per slice. Conduct puncture tests on individual vegetable slices.

Shear characteristic testing method: Select slices of cooked vegetables for shear testing. The slices should be taken from the middle section of the vegetable, which is the edible part, and should be approximately 0.5 cm thick. Cut the slices longitudinally to avoid holes. Prior to testing, select vegetable slices that are relatively uniform in size, shape and colour. Discard any slices that show significant damage after cooking.

Modifications were made to the Exponent software Sample Project method. Experiments were conducted using the HDP/BS flat-blade tooling assembly. Test mode: Compression. Options: Return to Start. Pre-test speed: 10.0 mm/s. Test speed: 1.0 mm/s. Post-test speed: 15.0 mm/s. Target mode: Distance. Test distance: 4.0 mm. Trigger type: Force. Trigger force: 5.0 g. Data acquisition rate: 200 pps.

Prior to testing, repeated ‘test cutting’ operations were performed to ensure there was no friction between the tool and the tool slot. The observed parameters were shear strength, shear energy and number of peaks.

Each treatment group was tested at least 15 times to minimize error.

### 2.3. Participants and Ethics

#### 2.3.1. Determination of Characteristic Values for Oral Processing Behaviour

Healthy females aged 18 to 27 years were recruited and screened. The inclusion criteria were as follows: body mass index (BMI) between 18.5 and 23.9 kg/m^2^; stable body weight over the preceding two months and no weight-loss dieting within the past year; absence of oral diseases or tooth sensitivity, and no orthodontic appliances; regular meal times and sleep patterns; no history of allergy to potatoes or rice; absence of any digestive system disorders or gastrointestinal discomfort; non-smoker and non-drinker; no metabolic disease; no eating disorders. Written informed consent was obtained from all participants. They were required to maintain regular eating patterns on the day before each trial session. On the trial day, they consumed a standard breakfast at a fixed time and avoided staying up late.

#### 2.3.2. Staple Food Substitution Trial

Potential participants were recruited through social media advertisements and posters on campus. The inclusion criteria were as follows: healthy female university students with a BMI between 18.5 and 23.9 kg/m^2^; regular sleep–wake cycles; a habit of eating breakfast regularly; consistent mealtimes throughout the day; and no allergic reactions to any of the test foods.

The exclusion criteria used to screen potential participants were as follows: presence of metabolic diseases; smoking or alcohol consumption habits; current participation in professional athletic training; abnormal liver or kidney function; mental or psychological disorders; eating disorders such as anorexia nervosa, bulimia nervosa, or binge eating disorder; long-term medication use or drug dependence; sleep disorders including insomnia, sleep apnea, circadian rhythm disorders, or restless legs syndrome; digestive disorders or frequent gastrointestinal discomfort; and extreme eating tendencies, as assessed by the Dutch Eating Behavior Questionnaire (DEBQ).

Following the initial screening, eligible participants were required to visit the laboratory prior to the formal experiment to undergo baseline measurements, including height, weight, body fat percentage, resting metabolic rate (RMR), waist circumference, and hip circumference. The HBF-371 body composition analyzer was used to determine RMR, body weight, and body fat percentage. Resting blood pressure was measured twice using an electronic blood pressure monitor, while waist and hip circumferences were measured using a tape measure. All willing participants underwent an oral glucose tolerance test (OGTT). Only those who met the following criteria and provided written informed consent were included in the study: fasting blood glucose < 6.1 mmol/L, peak blood glucose < 10.0 mmol/L, and 2 h blood glucose < 7.8 mmol/L. All tests were scheduled to avoid the menstrual period of female subjects. If menstruation occurred, the trial session was immediately terminated, and after the menstrual period ended, the affected trial sessions were repeated.

A statistical power analysis was performed using PASS 2021 software (NCSS, Kaysville, UT, USA). Based on the findings of Zhao et al. regarding potato [20], the minimum sample size for this study was determined to be 14 participants. This provides 80% power at a 5% significance level to detect differences in the incremental area under the curve (iAUC) for postprandial blood glucose, assuming a standard deviation (SD) of less than 18.4 mmol·min/L. To account for potential dropouts and other unforeseen circumstances, the recruitment target was increased to 20 participants.

All data were collected at the College of Food Science and Nutritional Engineering, China Agricultural University. The study protocol was designed in full accordance with the Declaration of Helsinki and was approved by the China Agricultural University Ethics Committee (Approval No.: CAUHR-20250501). The trial was also registered with the Chinese Clinical Trial Registry (Registration No.: ChiCTR2500104510, Clinical registration date: 18 June 2025).

### 2.4. Staple Food Substitution Study Design and Procedure

#### 2.4.1. Trial Design

The study adopted a randomized crossover design comprising three independent experimental treatments:

Control group with rice as the staple food (RC);

Hard-textured potato replacing one-third of the available carbohydrates from rice as the staple food (HP + R);

Soft-textured potato replacing one-third of the available carbohydrates from rice as the staple food (SP + R).

Each participant underwent three trials in a randomized sequence. A washout period of at least one day was observed between each treatment. Treatment sequences were randomly assigned using an online computer software program (http://www.randomizer.org, accessed on 10 June 2025). The experiment was scheduled to avoid coinciding with participants’ menstrual periods. If menstruation began or physical discomfort occurred, the session was rescheduled or terminated as appropriate. Participants were required to refrain from strenuous exercise and late nights, and to maintain a regular three-meal schedule on non-testing days throughout the trial. Due to ingredient limitations, blinding was not feasible.

The day before each trial, participants were instructed to eat three meals at 08:00, 12:00, and 18:00. They were also prohibited from consuming sugar-sweetened beverages, alcohol, or any other items that might introduce experimental error. They were required to fast after 20:00 and go to bed before midnight.

On the day of the experiment, participants arrived at the laboratory at 08:00 and consumed a standardized breakfast. They were instructed to engage only in light physical activities until lunch. At 12:00, they consumed the test meal at the experimental site, where data collection took place. Participants remained at the site until the end of the experiment at 21:00. From 08:00 on the day of the experiment until 08:00 the following day, participants were required to consume nothing except water.

#### 2.4.2. Postprandial Blood Glucose Monitoring

On each trial day, participants returned to the laboratory at 11:50 after having consumed breakfast. They rested for 10 min before baseline blood samples were collected. The test meal and 200 mL of water were then provided immediately afterward. Participants were instructed to finish the test meal within 5–10 min and to consume the water within 120 min of finishing the meal. Timing began with the first bite of food, defined as time 0 min. Postprandial fingertip blood glucose concentrations were measured at 0, 15, 30, 45, 60, 90, 120, 150, 180, 210, and 240 min.

**Figure 1:**
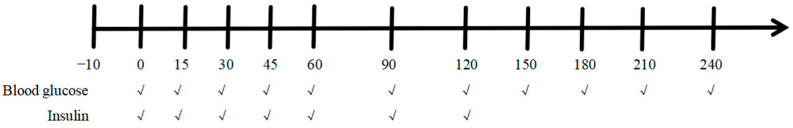
Time points for blood sample collection after the test meal. (√ was used to indicate that at this time point, blood glucose measurement or blood sample collection for insulin testing needs to be performed).

**Figure 2:**
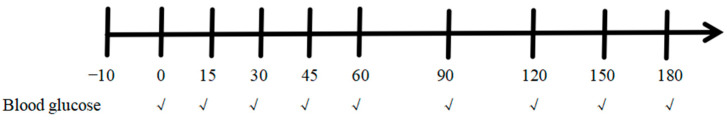
Time points for postprandial blood sampling after the second meal. (√ was used to indicate that at this time point, blood glucose measurement or blood sample collection for insulin testing needs to be performed).

At 18:00, participants were given a standardized dinner to finish within 10 min. This consisted of lettuce, cherry tomatoes, rice, salad dressing, luncheon meat, and chicken breast. Baseline indicators were collected at time 0, after which postprandial fingertip blood glucose concentrations were measured at 15, 30, 45, 60, 90, 120, 150, and 180 min. The specific time points are shown in Figure 1 and Figure 2.

Starting from the first bite of lunch, a scanner was used to record blood glucose changes from the continuous glucose monitoring sensor every 15 min until 180 min after dinner.

#### 2.4.3. Blood Sample Collection and Indicator Measurement

A 300 μL capillary blood sample was collected from each participant’s fingertip and transferred into an EDTA K_2_-treated anticoagulant centrifuge tube (WanDGL Ltd., Jinan, China) at baseline (0 min) before each lunch and at 15, 30, 45, 60, 90, and 120 min after meal consumption. Samples were temporarily stored at 4 °C. Within 30 min of collection, the samples were centrifuged at 1400× *g* for 10 min. Then, 60 μL of the resulting plasma supernatant was transferred into 0.5 mL Eppendorf tubes and stored at −80 °C until analysis. Plasma insulin concentration was determined using an ELISA-based assay kit (MEIMIAN Ltd., Yancheng, China).

#### 2.4.4. Oral Processing Behavior Measurement

Parameter measurement in this section was conducted simultaneously with the blood glucose response trial. The entire process was recorded via video camera, with participants required to maintain focused attention during eating, refraining from mobile phones use or conversation. The methodology for analyzing eating behavior was adapted from van Eck et al. [33]. The observed and recorded metrics included: total chews, eating duration, oral sensory exposure time, and chewing frequency. Specifically, “oral sensory exposure time” was defined as the cumulative duration from food entry into the mouth until swallowing. For the parameters of oral processing, ratings were conducted by three independent experimenters. Since the camera only captured the participants’ faces, the experimenters were unaware of the food being consumed, thereby establishing a blinding method. SPSS 27 was used to analyze reliability, and the intraclass correlation coefficient (ICC) value was 0.79, indicating satisfactory reliability.

#### 2.4.5. Assessment of Satiety

Satiety assessment was conducted alongside postprandial blood glucose measurements. For the first meal (lunch), satiety was evaluated at 0, 15, 30, 45, 60, 90, 120, 150, 180, 210, and 240 min after the start of the meal. For the second meal (dinner), satiety was assessed at 0, 15, 30, 45, 60, 90, 120, and 180 min after starting the meal. During the trial sessions, participants were prohibited from discussing, reading, or viewing any food-related content. They were also instructed not to communicate with each other about the test foods or their personal dietary sensations. Subjective perceptions of hunger, satiety, desire to eat, and prospective food consumption were quantified using the Visual Analogue Scale (VAS) method [34].

#### 2.4.6. Test Meal Details

The standard breakfast design is shown in Table 2. It consisted of 100 g of toast and 200 mL of pure milk, providing 400.9 kcal (56.87% from carbohydrates, 27.16% from fat, and 15.96% from protein).

The compositions for the different groups were as follows:

(1) RC (Control group): 90 g (raw weight) of cooked japonica rice served as the staple food.
(2) HP + R: 60 g (raw weight) of cooked japonica rice combined with 130 g (raw weight) of minimally cooked, firm-textured potato served as the staple food.
(3) SP + R: 60 g (raw weight) of cooked japonica rice combined with 130 g (raw weight) of thoroughly cooked, soft-textured potato served as the staple food.

The specific composition and macronutrient energy ratios of the test meals (lunch) are presented in Table 3 and Table 4. The energy sources and composition of the standardized dinner were identical to those of the control group (RC). To ensure the palatability, each participant consumed a test meal during a pre-trial session to confirm acceptability and ensure completion within the specified time.

### 2.5. Data Processing and Statistical Analysis

All glycemic indicators were analyzed based on the change from the fasting baseline. The incremental area under the curve (iAUC) for postprandial blood glucose and insulin responses above baseline was calculated using the trapezoidal rule. Glycemic variability parameters included postprandial peak blood glucose (ΔPeak_glu_), large amplitude glycemic excursion (LAGE), standard deviation of glucose (SD_glu_), coefficient of variation of glucose (CV_glu_), and the J-index, which was calculated as 0.324 × (mean blood glucose + SD_glu_)^2^ [35]. The LAGE was defined as the standard deviation of blood glucose differences at one-hour intervals within the observation period. The incremental area under the curve for postprandial insulin responses (iAUC_ins_) was also calculated. The postprandial insulin resistance index (HOMA-PP) was calculated as (iAUC_glu0–120_ × iAUC_ins0–120_)/22.5 [36], and insulin sensitivity was expressed using the Matsuda index, defined as 10,000/√(fasting glucose × fasting insulin × mean glucose × mean insulin) [37].

All statistical analyses were performed using GraphPad Prism version 8.3.0 for Windows (GraphPad Software, San Diego, CA, USA). Using the rice-based staple group as the control, multiple comparisons were adjusted using Tukey’s honestly significant difference (HSD) test, with statistical significance set at *p* < 0.05. A linear mixed-effects model was applied to evaluate differences between treatments and over time. Fixed effects include treatment effect, time effect, and treatment × time interaction effect, the random effect is a subject-level random intercept. Physicochemical indicators of the samples were analyzed using one-way analysis of variance (ANOVA) or the Kruskal–Wallis test with Bonferroni adjustment (*p* < 0.05). Physiological parameters of the subjects were analyzed using repeated-measures ANOVA followed by Duncan’s multiple range test; Friedman’s test was used for non-normally distributed results. Data are presented as mean ± standard error of the mean (SEM).

## 3. Results

### 3.1. In Vitro Digestibility Characteristics

Significant differences were observed in the in vitro starch digestive fractions among the tested meals, as presented in Table 5. The SP exhibited the highest rapidly digestible starch (RDS) content, while the HP had the lowest. The HP also showed the lowest slowly digestible starch (SDS) content, whereas the rice control (RC) had the highest. In terms of resistant starch (RS), the HP had the highest content, and the RC the lowest. Both the HP and SP had significantly higher RS contents than the RC. Additionally, the HP and SP differed significantly in RDS and RS contents (*p* < 0.0001).

### 3.2. Total Phenol Contents

**Figure 3:**
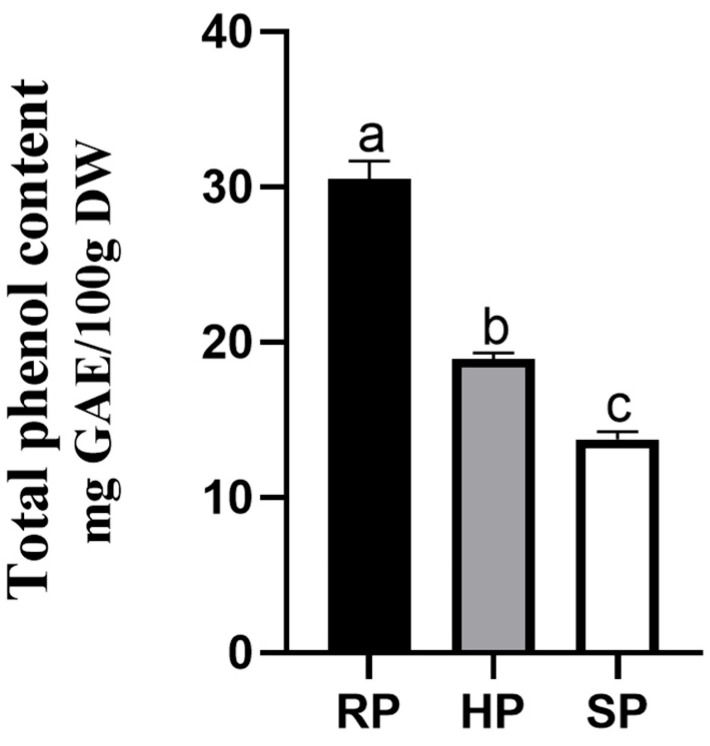
Total phenolic content in potatoes in each treatment group. a–c used for comparison between groups based on Kruskal–Wallis test with Bonferroni adjustment (*p* < 0.05). Values are mean ± SEM. RP, raw potato; HP, hard-cooked potato; SP, soft-cooked potato.

Figure 3 presents the total phenolic contents of the potato samples. The raw potato (RP) sample exhibited the highest total phenolic content, while the SP had the lowest. Compared to the raw sample, the total phenolic content of the minimally cooked HP decreased by 38%, whereas that of the fully cooked SP dropped by 55%.

### 3.3. Puncture Characteristics and Shear Characteristics

The puncture and shear characteristics of the potato samples are shown in Table 6. The raw sample exhibited the highest values for puncture strength, shear strength, shear energy, and shear brittleness by a considerable margin, while the SP group showed the lowest. Significant differences were observed among the raw sample, HP, and SP for all texture parameters except shear brittleness.

### 3.4. Baseline Characteristics of Participants of Staple Food Substitution Study

**Figure 4:**
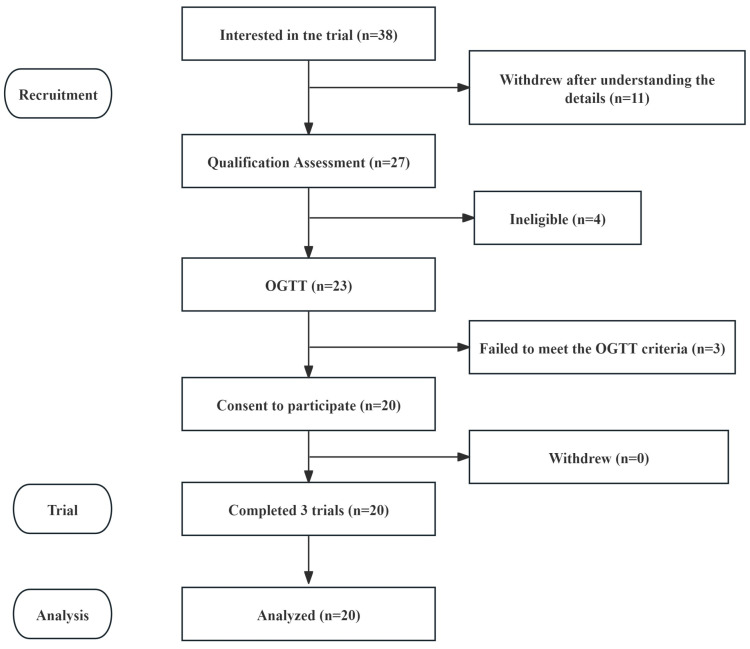
Flowchart of the Trial Procedure.

Figure 4 illustrates the flowchart of the study protocol. A total of 23 female volunteers initially underwent the oral glucose tolerance test (OGTT); however, three failed to meet the inclusion criteria and were excluded. Twenty participants completed the trial, and all data were included in the analysis. Baseline characteristics of the participants are summarized in Table 7. No adverse events or physical discomfort were reported by any participant throughout the study.

### 3.5. Postprandial Glycemic Responses to the Test Meal and the Second Meal

**Figure 5:**
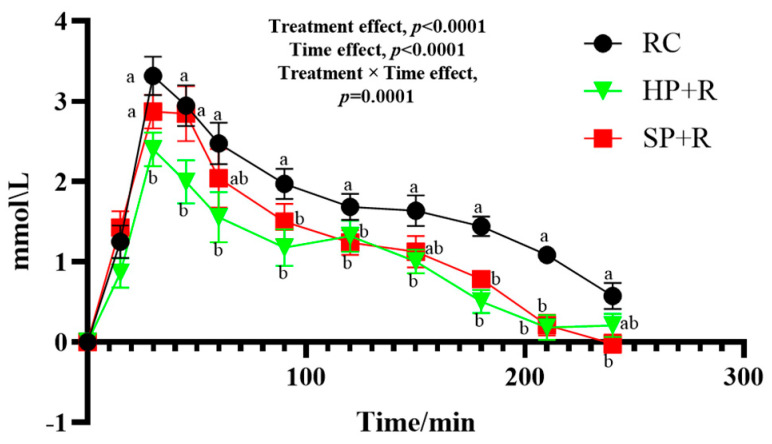
Postprandial glycemic response to test meals (finger-prick capillary glucose measurements). a, b, used for comparing glycemic level between groups at that time point based on repeated-measures linear mixed models, with Tukey adjustment (*p* < 0.05). The random intercept deviation is 0.194. Values are mean ± SEM. RC, rice control; HP + R, hard-cooked potato and rice; SP + R, soft-cooked potato and rice.

Figure 5 shows the postprandial glycemic response curves for the test meals. The HP + R and SP + R elicited significantly lower blood glucose levels than the rice control (RC) at all time points from 30 to 240 min and from 90 to 210 min, respectively. At 30 and 45 min, blood glucose levels in the HP + R group were significantly lower than those in the SP + R group.

Glycemic characteristics of the test meals are presented in Table 8. Compared with the rice control, the HP + R showed significantly lower values for all glycemic variability parameters except CV%. The SP + R, however, only exhibited a significantly lower iAUC_0–240_ and a higher CV% relative to the rice control. The HP + R also demonstrated significantly lower SD, J-index, and glucose peak values than the SP + R. Notably, the SP + R resulted in a 51.7% higher CV% than the rice control.

### 3.6. Postprandial Insulin Response to Test Meals

**Figure 6:**
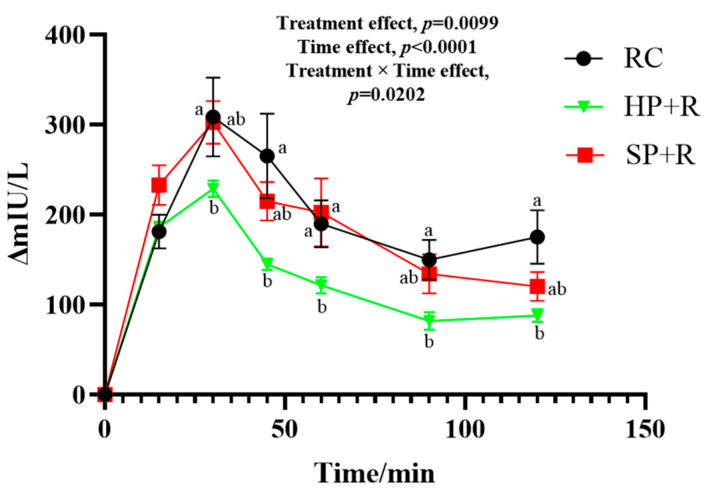
Postprandial insulin response to test meals. a, b, used for comparing insulin level between groups at that time point based on repeated-measures linear mixed models, with Tukey adjustment (*p* < 0.05). The random intercept deviation is 16.23. Values are mean ± SEM. RC, control group, rice as staple food; HP + R, hard potato and rice as staple food; SP + R, soft potato and rice as staple food.

The postprandial plasma insulin response to the test meals is shown in Figure 6. Overall, the insulin response to the SP did not differ from that of the rice control. In contrast, the HP + R resulted in significantly lower insulin levels from 30 to 120 min compared to the rice control, and also showed lower values at 30 and 60 min than the SP + R.

The characteristics of the insulin response are presented in Table 9. The HP + R exhibited a significantly improved insulin response compared to both the SP + R and the rice control in terms of iAUC_ins_, peak insulin level, SDins, and HOMA-PP. Furthermore, the HP + R enhanced insulin sensitivity relative to the rice control, whereas the SP + R did not.

### 3.7. Postprandial Glycemic Responses to the Second Meal

**Figure 7:**
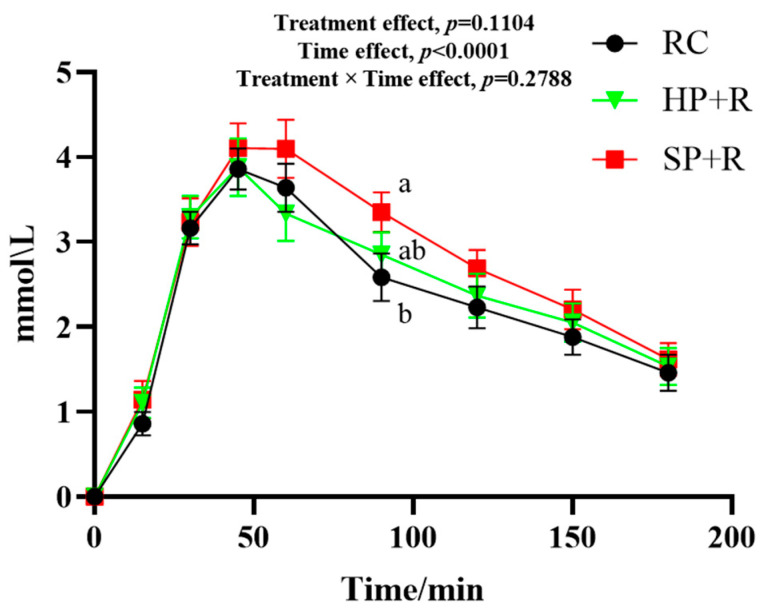
Postprandial glycemic response to the second meal (finger-prick capillary glucose measurements). a, b, used for comparing glycemic level between groups at that time point based on repeated-measures linear mixed models, with Tukey adjustment (*p* < 0.05). The random intercept deviation is 0.125. Values are mean ± SEM. RC, rice control; HP + R, hard-cooked potato and rice; SP + R, soft-cooked potato and rice.

Figure 7 shows the postprandial glycemic response curves for the second meal (standard dinner). At 90 min post meal, the blood glucose concentration in the SP + R group was significantly higher than that in the rice control group. No significant differences were observed between the HP + R and the rice control at any time point. Furthermore, no significant difference was found among the three groups in terms of glycemic variability parameters after the second meal.

**Figure 8:**
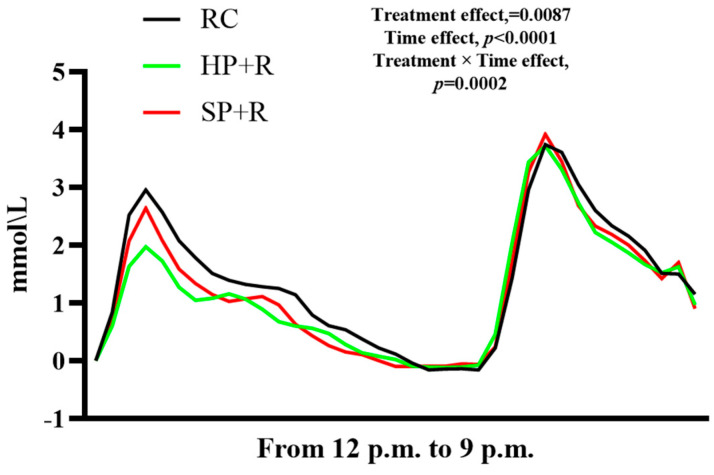
Postprandial glycemic response curve across both meals (CGM glucose measurements). The random intercept deviation is 0.131. RC, rice control; HP + R, hard-cooked potato and rice; SP + R, soft-cooked potato and rice.

**Figure 9:**
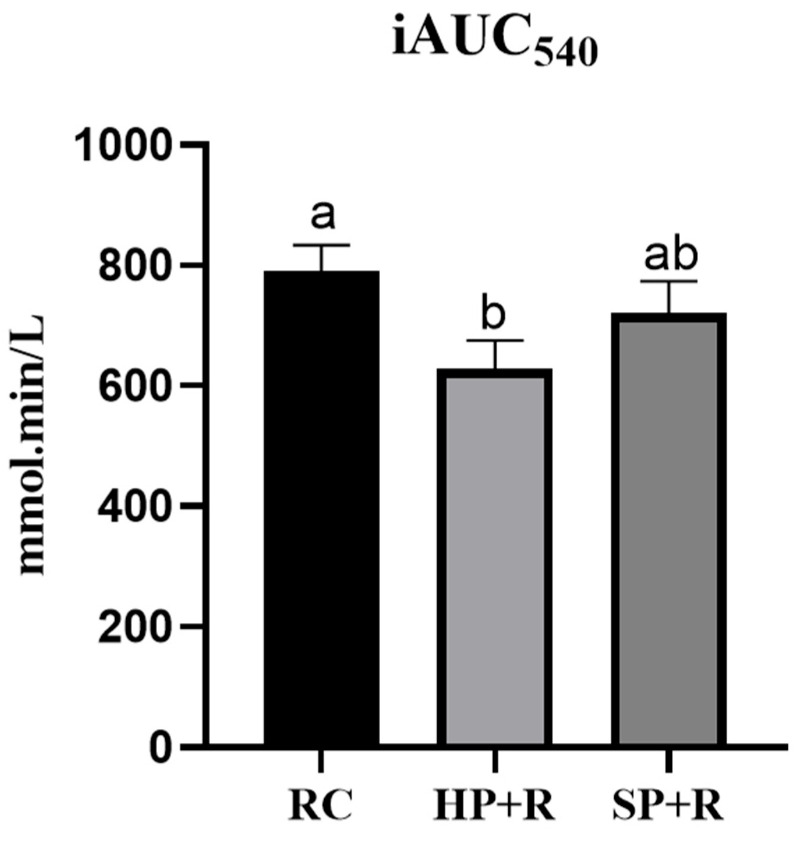
Total area under the glycemic response curve across both meals(CGM glucose measurements). a, b used for comparison between groups based on Friedman’s test (*p* < 0.05). RC, rice control; HP + R, hard-cooked potato and rice; SP + R, soft-cooked potato and rice.

Figure 8 presents the combined postprandial glycemic response curve across both the test and second meals, and Figure 9 shows the total iAUC_glucose_ over 540 min. The HP + R group achieved a significant reduction in total iAUC_glucose_ compared with the rice control, while no significant difference was observed between the SP + R and the rice control.

### 3.8. Characteristic Values for Oral Processing Behaviour

The characteristics of oral processing behaviors for the three staple foods are presented in Table 10. The HP + R elicited the highest number of total chews, followed by the rice control and the SP + R. Eating duration followed the order of HP + R, SP + R, and RC. The OSE time for the HP + R was significantly longer than that for both the SP + R and the RC. No significant difference in chewing frequency was observed between the SP + R and HP + R, although both were significantly lower than the RC in this regard.

Correlation analyses between glycemic characteristics and oral processing behaviors are shown in Table 11. Mean glycemic change exhibited a significant negative correlation with eating duration and a significant positive correlation with chewing frequency. Both iAUC_0–120_ and iAUC_0–240_ showed highly significant negative correlations with eating duration, as well as significant positive correlations with chewing frequency. These findings suggest that longer eating duration and lower chewing frequency are associated with attenuated postprandial glycemic responses.

Correlation analyses between insulin characteristics and oral processing behaviors are presented in Table 12. The iAUC_ins_ showed a significant negative correlation with total chews and eating duration. The postprandial insulin resistance index (HOMA-PP) demonstrated a highly significant negative correlation with eating duration. Peak insulin level was significantly negatively correlated with total chews. Therefore, longer eating duration and a greater number of chews are associated with a reduced insulin response and enhanced insulin sensitivity.

### 3.9. Postprandial Satiety Response to Test Meals

No significant difference was observed in satiety, hunger, food desire, or expected food intake among the three types of meals after either lunch or dinner.

## 4. Discussion

The present study investigated the associations among starch fractions, texture, oral processing behavior, and glycemic responses of cooked potatoes. In this acute trial, the effects of minimally cooked and fully cooled potatoes, incorporated as a one-third partial substitute for japonica rice, were evaluated. The mixed meal consisting of hard-cooked potatoes and rice (HP + R) significantly reduced postprandial glycemic and insulinemic responses, improved insulin sensitivity, and lowered the postprandial insulin resistance index compared with the rice control. Although the soft-cooked potato and rice mixture (SP + R) also significantly reduced the iAUC_0–240_ for glucose, it led to greater glycemic excursions and failed to improve insulin sensitivity. Consistent with our hypothesis 1 and 2, the degree of cooking did make differences to the glycemic and insulinemic properties of potatoes.

Previous studies have demonstrated that the in vitro digestible starch fraction of potatoes is highly dependent on cooking conditions [38]. Potato starch is particularly sensitive to heating duration, as evidenced by a sharp decline in resistant starch (RS) content from 87% in HP to 19% in SP, alongside a dramatic increase in rapidly digestible starch (RDS) from 9% in HP to 68% in SP. The core temperatures of the HP and SP samples in the present study were 57.6 °C and 77.4 °C, respectively. Given that the gelatinization temperature of potato starch is approximately 61.5 °C [39], the starch in the HP remained largely ungelatinized and thus indigestible. Zhao et al. [20] reported that microwave cooking for 2 min achieved approximately 70% RS retention in potato, a finding consistent with the results of the present study. These findings suggest that lightly cooked potato may serve as an economical, accessible, and practical source of daily resistant starch.

Despite comparable dietary fiber contents, the substantial differences in RS and RDS levels between the HP and SP likely played a major role in modulating the glycemic responses to the potato-rice mixed meals. The RS contents of HP + R, SP + R, and the rice control were 26.4 g, 11.6 g, and 6.1 g, respectively. These disparities in starch digestion patterns may partially explain why the SP + R failed to effectively attenuate postprandial blood glucose surges.

In the present study, it is noteworthy that the HP + R elicited significantly lower postprandial glucose and insulin responses, which may also be accompanied by potentially elevated insulin sensitivity, compared with the rice control. In contrast, the low-RS, high-RDS SP + R triggered a rapid elevation in blood glucose and induced a sharp insulin secretion, followed by a swift decline in glucose levels. However, the significant reduction in iAUC_0–240_ observed after the SP + R meal was not accompanied by improvements in insulin sensitivity or glycemic variability. It is widely accepted that RS can enhance insulin sensitivity by modulating the gut microbiota [40]; however, such microbiota-mediated effects typically require more than the 3 h postprandial period examined in this study. Therefore, the improved glycemic and insulin responses observed with HP + R are more likely attributable to the reduced bioavailability of starch from the high-RS HP in the mixed meal rather than to colonic fermentation of RS.

In addition to RS, the polyphenols present in potatoes may also contribute to improved postprandial glycemic profiles. The antidiabetic properties of polyphenols have been well documented in both in vivo and in vitro studies [41,42], and are mediated through multiple mechanisms, including inhibition of digestive enzymes, binding to carbohydrates, suppression of glucose transporters, modulation of insulin signaling pathways, attenuation of inflammatory responses, and protection of pancreatic β-cells from oxidative damage [43]. However, phenolic compounds are known to degrade substantially during prolonged heating [44]. The differing phenolic contents observed in the present study (19.0 mg GAE/100 g DW in HP vs. 13.7 mg GAE/100 g DW in SP) may thus have contributed to the divergent glycemic responses between the HP + R and SP + R meals.

In this study, texture and oral processing behaviors were also assessed. The lightly cooked HP retained a crispy and firm texture, whereas the fully cooked SP exhibited a soft texture. Accordingly, the HP sample demonstrated significantly higher values for puncture strength, shear strength, and shear energy compared with the SP sample. These textural differences likely influenced oral processing behavior [45], as reflected by the significantly greater total number of chews, longer eating duration, lower chewing frequency, and extended oro-sensory exposure (OSE) time observed in the HP + R group relative to the rice control.

Modified oral processing behaviors were reported to be associated with postprandial glycemic responses, possibly through alterations in food bolus particle size, kinetics of glucose release, salivary amylase activity, and insulin secretion [46,47]. Texture and oral processing may influence insulinemic responses by at least two pathways. On one hand, firmer food matrices tend to form larger boluses during mastication [46], which delays glucose release and absorption; on the other hand, reduced glucose availability may downregulate insulin secretion. Some studies have reported that more thorough chewing enhances insulin responses in healthy individuals [48] and in those at elevated risk for diabetes [46], suggesting that increased chewing and longer eating duration may promote early glucose uptake and insulin sensitivity. In another study, increasing the number of chews from 15 to 40 resulted in significantly higher insulin levels at the end of the meal [49]. In the present study, although insulin levels at 15–30 min were significantly higher in the SP + R group than in the HP + R group, glucose levels also remained elevated. This finding suggests that when digestible starch fractions differ substantially, oro-sensory exposure may not be the primary determinant of insulin response. Rather, the attenuated insulin response observed with HP + R is more likely attributable to its higher RS content and the consequent reduction in intestinal glucose release.

Regarding the second-meal glycemic response, no significant difference was observed between the two potato-containing meals and the rice control, either in terms of iAUC or glycemic variability. This finding suggests that the hypoglycemic effect of the HP did not persist into the subsequent meal.

The concept of the “second-meal effect” [24] refers to the phenomenon whereby consumption of low-glycemic index (GI) foods not only improves postprandial glucose tolerance following the first meal but may also attenuate the glycemic response to a subsequent meal. Previous studies have reported that certain low-GI foods, including cereals rich in resistant starch (RS) and β-glucan [50], as well as legumes such as chickpeas [51], may exert beneficial second-meal effects.

It is widely accepted that a high RS content plays a key role in mediating the second-meal effect [52]. Short-chain fatty acids (SCFAs) produced by colonic fermentation of RS [47] may act as signaling molecules, modulating gene expression and stimulating glucagon-like peptide-1 (GLP-1) secretion, thereby contributing to postprandial glycemic control [53]. For instance, consumption of RS-rich bagels has been shown to improve glucose and insulin homeostasis during both the first and second meals in individuals with diabetes [54].

However, despite its high RS content, the HP + R did not elicit a significant second-meal effect compared to the rice control in the present study. Similar findings have been reported, where refrigerated millet and rice with substantially increased RS contents following retrogradation failed to produce notable improvements in second-meal glycemic response [55]. We speculate one possible reason for this result is that, as RS may require considerable time to reach the distal intestine and undergo fermentation [56], the three- to six-hour intermeal interval may be insufficient for fermentation-related effects to manifest. In the aforementioned study [55] and the present investigation, the intervals between the test meal and the second meal were 3 and 6 h, respectively. This suggests that RS and other fermentable components may not be the sole contributors to the second-meal effect. While RS may exert significant glucose-lowering effects during the first meal, its fermentation-dependent metabolic benefits may not become observable until the following morning. Indeed, a prior study found that high intake of fermentable carbohydrates (e.g., pasta) at dinner significantly reduced the glycemic response to a standardized breakfast the next morning [52].

Alternatively, slowly digestible starch (SDS) may contribute more substantially to the second-meal effect within a several-hour timeframe. A previous study demonstrated that high-SDS corn starch elicited a significant second-meal effect [57] and was associated with elevated GLP-1 levels between 3 and 6 h after meals [58], which fits into the time window that aligns closely with the timing of the second meal in such study designs. Consistent with this hypothesis, the low SDS content of the HP in the present study may have precluded it from exerting a detectable second-meal effect.

Furthermore, we hypothesize that circadian rhythms may also be a contributing factor to this outcome, in previous studies on the second-meal effect, lunch was typically employed as the second meal, whereas the present study utilized dinner for this purpose. It is well established that both insulin secretion and insulin sensitivity exhibit circadian rhythms, peaking in the morning and declining progressively thereafter [59]. Gastric emptying rate also tends to decrease from morning to evening, accompanied by reduced small intestinal motility [60]. Consequently, the delivery of SDS and RS to the distal intestine and colon may require more time in the present study, thereby necessitating extended fermentation and a prolonged period for GLP-1 production. Given that the insulinotropic effect of GLP-1 is glucose concentration-dependent, by the time GLP-1 is induced via RS fermentation, postprandial glycemic levels following the second meal may have already declined to a relatively low baseline, thereby diminishing the opportunity for GLP-1 to elicit a pronounced second-meal effect. Moreover, the diminished insulin sensitivity in the evening, which contributes to relatively greater postprandial glycemic excursions, may further obscure any modest second-meal effect [59]. However, the incretin response including GLP-1 across the two meals was not assessed in this study. Therefore, the above proposed association needs to be verified in future research. For instance, the real time process of RS fermentation can be assessed by breath hydrogen measurement [61] and microbial-derived metabolites in blood [62].

This study is the first randomized controlled trial to examine the effects of cooked potatoes on oral processing behavior, postprandial insulin responses, and second-meal effects. Potatoes were used as partial substitutes for rice, which was in line with the typical consumption pattern in daily life. Two cooking procedures were employed to simulate stir-frying and stewing, which resulted in different levels of RS. The textural properties and oral processing characteristics of the processed potatoes were examined, the proportions of RDS, SDS and RS were measured, and potential associations between starch fractions and first- and second-meal glycemic responses were explored.

Several limitations of the present study should be acknowledged. First, the study participants were exclusively young, healthy women, which limits the generalizability of the findings to males, individuals with diabetes, or older populations. Second, due to participant compliance constraints, GLP-1 levels in response to the test meals were not measured, thereby restricting the exploration of potential relationships between glycemic responses and oral processing parameters. Additionally, blood samples for insulin and incretin assays were not collected after the second meal, which limits the ability to interpret the hormonal patterns underlying the second-meal glycemic response. Third, future studies incorporating hydrogen breath tests, along with metabolomic analysis of plasma and fecal samples, would help verify the time course of resistant starch fermentation and its potential role in mediating the second-meal effect. Fourth, as an acute meal study, the present findings warrant further validation through long-term intervention trials.

## 5. Conclusions

In summary, consistent with part of our hypotheses, this study demonstrates that replacing one-third of the rice in healthy female subjects’ diets with minimally cooked potatoes attenuated their postprandial glycemic and insulinemic responses. The glycemic variability was mitigated in terms of lowered peak glucose levels, decreased iAUC, and reduced glycemic excursion, and the insulin sensitivity may be improved in terms of HOMA-PP and Matsuda index. Rich RS content and reduced glucose availability, rather than the texture and enhanced oral processing, may underpin the favorable glycemic and insulin effect of HP + R.

However, the glycemic impact of HP + R did not persist into the second meal. The study suggests that the second-meal effect was not only impacted by RS content and we speculate a few hours might not be enough for effective colonic fermentation and SCFA production needed for stabilizing the glucose level of the subsequent meal.

While the mechanisms by which cooked potatoes impact glycemic behaviors, the second-meal effect and its universality across different populations require further research; we hope the findings in this study provide a new perspective for potato-consumption patterns conducive to easy and effective blood glucose management.

## Figures and Tables

**Table 1:** Physicochemical composition of starchy vegetables and Japonica rice (g/100 g).

Ingredients	Moisture	Available Carbohydrates ^1^	Fat	Protein	Dietary Fiber	Starch	Ash
Potato	77.08	17.87	0.3	1.8	2.2	10	0.75
Japonica rice	14	77.2	0.6	6.6	0.7	77.2	0.9

^1^ Available Carbohydrate Content (%) = 100% − Moisture Content (%) − Fat Content (%) − Protein Content (%) − Dietary Fiber Content (%) − Ash Content (%).

**Table 2:** Macronutrient Composition of the Standard Breakfast.

Food	Carbohydrates/g	Fat/g	Protein/g	
100 g toast	47.4	4.5	9.6	
200 mL Whole Milk	9.6	7.6	6.4	
Total Energy/kcal	228	108.9	64	400.9
Energy Distribution	56.87%	27.16%	15.96%	

**Table 3:** Main Nutrient Composition of the Test Lunch and dinner.

Test Meal	Carbohydrate from Rice (g)	Carbohydrate from Potatoes (g)	Carbohydrate from Other Food (g)	Total Carbohydrate (g)	Protein (g)	Fat (g)	Fiber (g)	Energy (kcal)
Lunch (R ^1^)	69.5	-	10.0	79.5	21.8	17.0	0.63	558.3
Lunch (P ^1^)	46.3	23.2	10.0	79.5	22.0	17.4	3.28	562.9
Dinner	69.5	-	10.0	79.5	21.8	17.0	--	558.3

^1^ R represents the rice staple group, P represents the potato partial substitution staple group.

**Table 4:** Macronutrient energy contribution ratios and details of the test meals.

Test Meal	Energy from Carbohydrate	Energy from Protein	Energy from Fat	Detail
Lunch (R ^1^)	56.96%	15.59%	27.44%	uncooked rice 90 g, instant chicken breast 40 g, roasted sesame dressing 25 mL, lettuce 40 g, luncheon meat 35 g, cherry tomato 50 g
Lunch (P ^1^)	56.50%	15.66%	27.84%	uncooked rice 60 g, uncooked potato 130 g, instant chicken breast 40 g, roasted sesame dressing 25 mL, lettuce 40 g, luncheon meat 35 g, cherry tomato 50 g
Dinner	56.96%	15.59%	27.44%	uncooked rice 90 g, instant chicken breast 40 g, roasted sesame dressing 25 mL, lettuce 40 g, luncheon meat 35 g, cherry tomato 50 g

^1^ R represents the rice staple group, P represents the potato partial substitution staple group.

**Table 5:** In Vitro Digestibility Characteristics of Each Treatment Group. a–c used for comparison between groups based on Kruskal–Wallis test with Bonferroni adjustment (*p* < 0.05). R, rice; HP, hard-cooked potato; SP + R, soft-cooked potato.

	RDS (%)	SDS (%)	RS (%)
R	36.8 ± 3.5 ^b^	53.8 ± 5.3 ^a^	9.4 ± 2.5 ^c^
HP	9.4 ± 0.8 ^c^	3.4 ± 1.2 ^c^	87.2 ± 2.0 ^a^
SP	68.1 ± 4.6 ^a^	12.5 ± 3.0 ^b^	19.4 ± 4.6 ^b^
HP + R ^1^	30.8	42.8	26.4
SP + R ^1^	43.7	44.7	11.6

^1^ represents the calculated proportion of each starch component in the staple food substitute meal. The corresponding proportion in the staple food substitute meal = (The proportion of this type of starch in potatoes × 13 (starch content in 130 g of potatoes) + the proportion of this type of starch in japonica rice × 46.32 (starch content in 60 g of japonica rice))/(13 + 46.32).

**Table 6:** Puncture and shearing characteristics of potato in each treatment group. a–c used for comparison between groups based on Kruskal–Wallis test with Bonferroni adjustment (*p* < 0.05). Values are mean ± SEM. RP, raw potato; HP, hard-cooked potato; SP, soft-cooked potato.

	Puncture Strength/g	Shear Strength/N	Shear Energy/(N·mm)	Shear Peak
RP	574.5 ± 14.1 ^a^	63.6 ± 2.5 ^a^	206.0 ± 13.0 ^a^	5.6 ± 0.7 ^a^
HP	385.3 ± 10.9 ^b^	22.8 ± 0.8 ^b^	82.6 ± 4.2 ^b^	3.9 ± 0.3 ^ab^
SP	63.8 ± 5.5 ^c^	1.5 ± 0.1 ^c^	3.9 ± 0.3 ^c^	2.3 ± 0.2 ^b^

**Table 7:** Baseline Characteristics of Participants.

Characteristics	Mean ± SEM
Age (years)	21.2 ± 0.3
Body composition	
BMI (kg/m^2^)	20.2 ± 1.2
Fat mass (%)	26.8 ± 0.8
Waist: hip ratio	0.8 ± 0.1
Visceral fat index	3.1 ± 0.3
Resting metabolic rate (kcal/day)	1251.0 ± 18.6
Physical examination	
Systolic blood pressure(mmHg)	105.0 ± 2.3
Diastolic blood pressure (mmHg)	65.7 ± 1.7

**Table 8:** The Postprandial Glycaemic Characteristic Parameters of Test Meals. a, b used for comparison between groups based on Friedman’s test (*p* < 0.05). RC, rice control; HP + R, hard-cooked potato and rice; SP + R, soft-cooked potato and rice.

	Mean Change/ (mmol/L)	LAGE/ (mmol/L)	SD/ (mmol/L)	CV/%	J-Index	ΔPeak (mmol/L)	iAUC_0–120_ (mmol·min/L)	iAUC_0–240_ (mmol·min/L)
RC	1.7 ± 0.1 ^a^	3.7 ± 0.2 ^a^	1.1 ± 0.1 ^a^	69.2 ± 3.7 ^b^	19.6 ± 0.9 ^a^	3.6 ± 0.2 ^a^	252.7 ± 17.6 ^a^	412.0 ± 27.7 ^a^
HP + R	1.0 ± 0.1 ^b^	3.0 ± 0.2 ^b^	1.0 ± 0.1 ^b^	88.5 ± 14.4 ^ab^	16.7 ± 0.8 ^b^	2.7 ± 0.2 ^b^	171.4 ± 20.5 ^b^	256.3 ± 30.7 ^b^
SP + R	1.3 ± 0.1 ^ab^	3.7 ± 0.3 ^ab^	1.2 ± 0.1 ^a^	105.6 ± 10.4 ^a^	18.9 ± 1.2 ^a^	3.4 ± 0.3 ^a^	217.1 ± 21.1 ^ab^	305.2 ± 26.7 ^b^

**Table 9:** Insulinemic Characteristic Parameters of Test Meals. a, b used for comparison between groups based on Friedman’s test (*p* < 0.05). RC, control group, rice as staple food; HP + R, hard potato and rice as staple food; SP + R, soft potato and rice as staple food.

Test Meal	iAUC_ins_ (mIU × min/L)	HOMA-PP (×10^3^)	Insulin Sensitivity	Peak_ins_ (mIU/L)	SD_ins_
RC	23,224 ± 2456 ^a^	266.6 ± 34.4 ^a^	10.1 ± 0.6 ^b^	358.2 ± 51.7 ^a^	123.6 ± 16.6 ^a^
HP + R	14,517 ± 801 ^b^	110.9 ± 14.7 ^b^	12.9 ± 0.5 ^a^	234.0 ± 7.1 ^b^	80.3 ± 8.1 ^b^
SP + R	21,635 ± 1829 ^a^	220.2 ± 30.4 ^a^	12.2 ± 0.8 ^ab^	357.9 ± 32.5 ^a^	123.9 ± 10.6 ^a^

**Table 10:** Oral processing behavior parameters in different trial groups. a–c used for comparison between groups based on Kruskal–Wallis test with Bonferroni adjustment (*p* < 0.05). Values are mean ± SEM. RC, rice control; HP + R, hard-cooked potato and rice; SP + R, soft-cooked potato and rice.

	Total Chews	Eating Duration/s	OSE Time/s	Chewing Frequency (Times/s)
RC	871.1 ± 52.2 ^b^	530.1 ± 57.4 ^c^	524.3 ± 47.7 ^b^	1.7 ± 0.1 ^a^
HP + R	1012.6 ± 60.4 ^a^	777.6 ± 68.2 ^a^	566.5 ± 64.6 ^a^	1.3 ± 0.1 ^b^
SP + R	808.1 ± 55.9 ^c^	638.4± 61.9 ^b^	515.4 ± 54.9 ^b^	1.3 ± 0.1 ^b^

**Table 11:** Correlation between glycemic characteristics and Oral processing behavior parameters (*n* = 20).

		OSE	Total Chews	Eating Duration	Chewing Frequency	Mean Change	iAUC_0–120_	iAUC_0–240_
OSE	Pearson correlation	1						
Total Chews	Pearson	0.812 **	1					
Eating Duration	Pearson correlation	0.132	0.341 **	1				
Chewing Frequency	Pearson correlation	0.428 **	0.411 **	−0.687 **	1			
Mean Change	Pearson correlation	0.055	−0.071	−0.394 **	0.342 **	1		
iAUC_0–120_	Pearson correlation	−0.002	−0.12	−0.328 *	0.257 *	0.955 **	1	
iAUC_0–240_	Pearson correlation	0.085	−0.038	−0.409 **	0.379 **	0.986 **	0.939 **	1

** Significant at the 0.01 level (two-tailed). * Significant at the 0.05 level (two-tailed).

**Table 12:** Correlation between insulin characteristics and Oral processing behavior parameters (*n* = 20).

		OSE	Total Chews	Eating Duration	Chewing Frequency	iAUC_ins_	HOMA-PP	Peak_ins_
OSE	Pearson correlation	1						
Total Chews	Pearson correlation	0.812 **	1					
Eating Duration	Pearson correlation	0.132	0.341 **	1				
Chewing Frequency	Pearson correlation	0.428 **	0.411 **	−0.687 **	1			
iAUC_ins_	Pearson correlation	−0.118	−0.259 *	−0.260 *	0.076	1		
HOMA-PP	Pearson correlation	−0.09	−0.235	−0.341 **	0.186	0.851 **	1	
Peak_ins_	Pearson correlation	−0.184	−0.296 *	−0.227	0	0.853 **	0.707 **	1

** Significant at the 0.01 level (two-tailed). * Significant at the 0.05 level (two-tailed).

## Data Availability

The datasets presented in this article are not readily available due to privacy. Requests to access the datasets should be directed to the corresponding author.

## Abbreviations

The following abbreviations are used in this manuscript:

AC	Available carbohydrate
GI	Glycaemic index
OGTT	Oral glucose tolerance test
RS	Resistant starch
RDS	Rapidly digestible starch
SDS	Slowly digestible starch
R	Rice
RP	Raw potato
HP	Hard potato
SP	Soft potato
RC	Control group, rice as staple food
HP + R	Hard potato and rice as staple food
SP + R	Soft potato and rice as staple food
IAUC	Incremental area under the curve
CONGA1_glu_ and CONIA1_ins_	Consecutive 1 h intervals of net glucose/insulin action
